# Hox Targets and Cellular Functions

**DOI:** 10.1155/2013/738257

**Published:** 2013-12-30

**Authors:** Ernesto Sánchez-Herrero

**Affiliations:** Centro de Biología Molecular Severo Ochoa (CSIC-UAM), Nicolás Cabrera 1, Universidad Autónoma de Madrid, Cantoblanco, 28049 Madrid, Spain

## Abstract

Hox genes are a group of genes that specify structures along the anteroposterior axis in bilaterians. Although in many cases they do so by modifying a homologous structure with a different (or no) Hox input, there are also examples of Hox genes constructing new organs with no homology in other regions of the body. Hox genes determine structures though the regulation of targets implementing cellular functions and by coordinating cell behavior. The genetic organization to construct or modify a certain organ involves both a genetic cascade through intermediate transcription factors and a direct regulation of targets carrying out cellular functions. In this review I discuss new data from genome-wide techniques, as well as previous genetic and developmental information, to describe some examples of Hox regulation of different cell functions. I also discuss the organization of genetic cascades leading to the development of new organs, mainly using *Drosophila melanogaster* as the model to analyze Hox function.

## 1. Introduction

The amazing variety of animal forms has always attracted the curiosity of scientists and spurred investigations into the underlying cause of such diversity. Comparison of different species, or of serially similar parts within the same animal, led to the concept of homology and to the idea that a basic pattern of development could be variously modified to obtain different structures. Part of the explanation for such diversity, particularly that of serially homologous organs, relies on the activity of Hox genes. These genes have received other names in the past: selector genes or master genes, indicating that they direct a particular developmental pathway, or homeotic genes, because mutations in them frequently cause transformation of one part of the body into another, that is, homeosis. However, these names include genes that do not meet all the characteristics that define Hox genes, as explained below.

Hox genes are a group of genes conserved in evolution that determines the development of different structures along the anteroposterior (A/P) axis of bilaterians [1, 2]. They are usually clustered in gene complexes, although conservation of clustering in evolution is more evident in chordates [3–5]. These genes have been studied in many species, but with more detail in two of them, *Drosophila melanogaster* and mouse. In *Drosophila melanogaster*, Hox genes are grouped in two complexes, the bithorax complex (BX-C), comprising the Hox genes *Ultrabithorax* (*Ubx*), *abdominal-A* (*abd-A*), and *Abdominal-B* (*Abd-B*) [3, 6–8] and the Antennapedia complex (ANT-C), including the Hox genes *labial* (*lab*), *proboscipedia* (*pb*), *Deformed* (*Dfd*), *Sex combs reduced* (*Scr*), and *Antennapedia* (*Antp*) [9, 10]. In the mouse there are four complexes including the paralogous Hox genes 1 to 13, to a total of 39, although no single complex has the whole repertoire of Hox genes [11–13]. These genes are highly conserved in evolution, and there is a correspondence between particular Hox genes in *Drosophila* and mouse (and other species) [11, 12, 14]. As in *Drosophila*, one of the main functions of Hox genes in the mouse is to establish the A/P axis, thus specifying the development of different elements of the axial skeleton, although there is also an important function of Hox genes in limb development [15, 16].

The expression of Hox genes along the A/P axis relates to their position within the cluster. Genes at one end of the cluster are expressed more anteriorly and those at the other end more posteriorly, a phenomenon known as spatial colinearity [3, 17]. In vertebrates, there is also a temporal sequence of activation of Hox genes, so that 5′ genes are expressed later and more posteriorly in the A/P axis, whereas those located at the other end of the complex are expressed earlier and more anteriorly [18, 19].

Hox genes code for proteins that bind DNA and regulate the expression of different targets. Hox proteins include a highly conserved sequence-specific DNA-binding domain, the homeodomain. The analysis of Hox protein binding activity *in vitro* identified a short DNA motif bound by the different proteins, with low discrimination between them, although preferences in sequence recognition have been described [20–23]. The relatively unspecific recognition of sequences by Hox proteins posed the problem of how they could bind similarly *in vitro* but determine different structures *in vivo*.

Part of the solution of the paradox relies on the fact that Hox proteins do not act alone but are aided by different cofactors and collaborators [23]. The best-characterized cofactors belong to the TALE class of homeoproteins and are encoded by *Pbx* and *Meis* genes in vertebrates (*extradenticle* and *homothorax*, resp., in *Drosophila*). The coexpression in the nucleus of Pbx/Exd (and Meis/Hth) and a particular Hox protein increases Hox specificity and affinity for downstream targets [24, 25]. Thus, the collaboration of Exd with different Hox proteins in *Drosophila* determines the specific recognition of certain sequences and, therefore, provides a means of selecting particular targets for each Hox protein [23, 26, 27].

Hox genes were initially identified in *Drosophila* by the spectacular transformations (homeosis) observed in Hox mutations [28–30], although homeotic transformations were first described in other species [31]. In many *Drosophila* Hox mutations, whole segments or structures are transformed into the like of another one within the *Drosophila* body. For instance, mutations in the *Ultrabithorax* (*Ubx*) gene transform the third thoracic segment (T3) into the second one (T2), including the development of four wings in the thorax instead of the wildtype pattern of two wings and two halteres (small dorsal appendages of the T3 needed to fly) [30]; similarly, gain-of-function mutations in *Antennapedia* transform the antennae into legs [32–34]. In the mouse, the existence of paralogous Hox genes in four different complexes prevents complete transformations when a single Hox gene is inactivated, and only partial transformations along the anteroposterior axis were initially reported [35–37]. However, when all the paralogous Hox genes from the different clusters were simultaneously deleted, complete transformations in the axial skeleton occur [37–39]. Similarly, simultaneous inactivation of Hox genes from different paralog groups frequently leads to phenotypes that are a combination of those of single mutations, although more complex phenotypes are also observed [1, 3, 15].

Such transformations require major changes in the number and properties of cells, such as cell division rates, cell affinities, and cell differentiation. This implies that Hox genes must regulate many genes responsible for cellular functions to elicit the wildtype pattern. Moreover, there must be a coordination in these changes, since the result is the harmonious development of a new structure or organ. Even in the cases in which Hox mutations do not produce clear transformations, regulation of downstream targets is usually quite specific. Because Hox genes are expressed in different tissues, the changes occur in different cell types and require the regulation by Hox proteins of distinct sets of downstream genes. Unraveling how Hox genes regulate cellular functions through specific sets of targets not only it is indispensable for understanding the role of these genes in development but also it has implications in other fields of biology. For example, it was assumed early on that changes in Hox gene expression or activity could account for some morphological changes in evolution [3], and elucidating how different regulation of Hox targets is achieved may explain the generation of new forms.

In this review, I will describe some of the cellular functions performed by Hox genes and the targets they regulate. Although effects of Hox genes have been reported in derivatives of the ectoderm, mesoderm, and endoderm, and in both invertebrates and vertebrates, the main focus will be in the development of epidermal structures and the generation of particular organs or structures. Since the organism where this analysis is more advanced is *Drosophila*, the fruit fly will be the predominant target of our analysis.

## 2. Search for the Targets

The classical hypothesis of selector genes of García-Bellido [40] proposed that Hox genes (selector genes at that time, including also genes like *engrailed*, determining the posterior compartment of segments) acted by regulating a series of targets, called “realizator" genes, which would carry out the critical roles needed to organize different organs. The term “realizator” meant any gene directly connected with basic cellular functions, but it was not precisely defined. Realizator genes would be those required to determine distinct cell affinities, regulate cell proliferation, and establish organ shape [40]. The identification of realizator genes was, therefore, key to understand how Hox genes shape different structures. Different approaches to isolate and characterize Hox targets in *Drosophila* and vertebrates were initially used to try and identify such genes, including chromatin immunoprecipitation, UV cross-linking and DNA immunoprecipitation, subtractive hybridization, antibody staining in salivary glands, one-hybrid screening in yeast, and analysis of different patterns of expression along the A/P axis (reviewed in [23, 41–49]). Many of the targets were identified just by observing their different segmental expression in the embryo and their response to mutations in Hox genes, and only a few were shown to be directly regulated by Hox genes (see comprehensive lists in [44, 46, 47, 49]). Interestingly, many of the Hox targets proved to be genes coding for transcription factors themselves, suggesting that the architecture of Hox regulation relied on intermediate factors that would subdivide tasks. However, a few of the direct targets first identified, such as centrosomin [50] or connectin [51], conformed to the definition of realizator genes.

More recently, genome-wide techniques such as microarrays [52–73] and chromatin immunoprecipitation (ChIP) [74–80] have been used to determine the genes bound or regulated by Hox proteins in *Drosophila*, mouse, or cultured cells (Table 1). These methods have shown that hundreds of genes are downstream Hox targets.

### 2.1. Differential Expression Studies

The first attempt to identify Hox gene targets in *Drosophila* with microarray technology was done by comparing gene expression in wildtype embryos and in embryos after uniform expression of the Hox gene *lab* [53]. The *lab* gene of the ANT-C is expressed in the head and is required for the development of some head structures [81–83]. The authors probed 1513 genes and found 96 with significant changes in expression levels between the two types of embryos. Among the *lab* downstream genes identified, the most common class belonged to the transcriptional regulation group, followed by the metabolism, proteolytic system/apoptosis, and cell surface receptors (cell adhesion molecules) groups. Interestingly, within the proteolytic systems/apoptosis class, 12 out of 13 genes were upregulated in the embryos overexpressing *lab*, within the cell surface regulators/CAMs/ion changes class, 10 out of 12 were downregulated, and in the cell cycle and transcription/replication/repair class, all the genes differentially expressed in wildtype and *lab*-overexpressing embryos were upregulated. This suggests a similar regulation by *lab* of the elements involved in a particular cellular process.

Since Hox genes differentiate between homologous segments, changes in Hox downstream gene transcription are expected between segments with different Hox expression. Following this logic, two studies [54, 61] compared gene expression between the *Drosophila* leg discs. The three pairs of leg discs, located in the prothoracic (T1), mesothoracic (T2), and metathoracic (T3) segments, express different Hox genes and give rise to the three pairs of legs, with roughly similar shape and size, but differentiating specific patterns. In the first work [54], the authors found 2 genes differentially expressed in T1 and T3 leg discs, 12 when comparing T2 and T3 discs and 17 in T1 and T2 discs. In the second study [61], the authors focused their analysis on the genes specifically expressed in the T1 leg disc. This disc, the only one requiring the expression of the Hox gene *Scr* [84, 85], differentiates the T1 leg, which shows some particular structures like the sex comb in males. Barmina and collaborators [61] analyzed 7125 genes expressed in the distal leg discs of 16 h old pupae, and, at a threshold *P* < 0,001, they found 34 genes differentially expressed between T1 and T2 leg discs, 10 of which coded for metabolic enzymes/transporters, 5 for transcription factors, and 5 for proteins of the extracellular ligand/receptor class. Therefore, many of the genes identified in these two studies [54, 61] code for transcription factors or for proteins involved in signaling pathways, but some of the genes are more directly involved in cellular functions.

Other microarray experiments analyzed the most studied homeotic transformation in *Drosophila*, the transformation of halteres into wings caused by mutations in *Ubx*. The halteres are the dorsal appendages of the third thoracic segment (T3) and the wings are homologous dorsal appendages of the second thoracic segment (T2). The structures of the dorsal T3 derive from the haltere disc, which expresses *Ubx*, whereas the dorsal T2 is formed from the wing disc, which lacks *Ubx* expression except for the peripodial membrane [86, 87]. Mutations in *Ubx* transform the haltere disc into the wing disc and, in the adult, T3 into T2, including the transformation of halteres into wings [30, 88]. Three studies have analyzed wing and haltere disc gene expression by using microarrays [64, 66, 73]. They used different approaches to identify genes differentially transcribed either in the whole wing and haltere discs or just in their pouch region (the region of the discs giving rise to the wings and halteres, resp.).

The first two studies [64, 66] identified many genes regulated by *Ubx*, although they could not distinguish direct from indirect targets. By contrast, a more recent microarray analysis made use of the Gal4/Gal80ts system [89] to induce *Ubx* at precise points of development in the wing disc and analyzed the immediate transcriptional response [73]. In this way, the authors could identify genes that were likely to be direct targets and that selectively responded to *Ubx* at different times of development. Their experiments proved that different genes are active at distinct points of larval and pupal development and that most of them respond to *Ubx* at one single stage. They also found, in a more stringent analysis, 308 *Ubx* targets, many of which can be considered as “realizator” genes since they are directly involved in diverse cellular functions: components of the cuticle and extracellular matrix, genes involved in cell specification, cell proliferation, cell survival, cell adhesion, or cell differentiation, structural components of the actin and microtubule filaments, and accessory proteins controlling filament dynamics. Only 10% of the genes identified in this work coincide with those of previous microarray analyses [64, 66] suggesting that many of the latter might not be under *Ubx* direct control. Finally, the authors also found that *Ubx *regulates many genes in a subtle way, with just small differences in levels of expression between the discs expressing or not this Hox protein.

A more extensive analysis of Hox target expression was carried out in the embryo by studying the genes responding to the ubiquitous expression of 6 out of the 8 Hox genes (the genes *pb* and *lab* were not analyzed) [67]. As also found in the study of Pavlopoulos and Akam [73] the authors discovered that different targets were activated at different time points, in this case stages 11 or 12 of embryonic development, suggesting a tight temporal control of target gene expression by Hox proteins. The immediate analysis by microarrays after the ectopic expression of Hox genes suggested that many of the genes identified were probably direct targets of Hox proteins (the authors calculate that they are 20%–30% of the total number). Hundreds of genes were found to respond to the ectopic expression of the different Hox genes and about 70% of them were regulated by only one Hox protein. It was also found that the major group of genes identified belonged to the category of realizators and that, within these, the most common group was formed by genes involved in proteolytic processes; other abundant groups, with decreased frequency, were those coding for cytoskeleton proteins, cuticle, chorion and peritrophic membrane, cell cycle and cell proliferation genes, genes involved in apoptosis, and genes coding for cell adhesion proteins [67]. 

Expression profile analyses have also been carried out in vertebrates or in cultured cells with different Hox activity [52, 55–60, 62, 63, 65, 68–72]. Some studies were done in cell lines mutant for a Hox gene or analyzing the transcriptional response after overexpressing a single Hox product [55, 57, 58, 60, 62]. Other experiments compared the transcription profile of a Hox mutant with that of the wildtype in a particular mouse [52, 56, 59, 63, 65, 71, 72] or zebrafish [68, 70] organ. As previously discussed [72], the choice of material is important since no overlap was found in the downstream targets of the same Hox gene (*Hoxa1*) in embryonic stem cells or developing embryos [60, 72]. Although in most cases the mutant condition analyzed caused strong transformations, one study compared the transcriptional response in the forelimb and genital eminence of wildtype mice and of mice heterozygous for a deletion in the HoxD locus, which produced just a mild phenotypic effect [59]. In this example, few genes were identified as differentially expressed in the mutant and the wildtype, suggesting a correspondence between this low number and the weak phenotype.

Another study compared gene expression in different wildtype rhombomeres [69], distinct anatomical domains of the vertebrate central nervous system that are specified by Hox genes [90]. The authors found that there were clear differences in transcription profiles in different rhombomeres but also discovered a uniform expression of some Hox targets, with just small differences between rhombomeres. This suggests that the Hox proteins may subtly modulate the transcription of targets and not simply dictate an on/off response. Interestingly, most of the targets were coding for proteins involved in cell communication, cell differentiation, cell death, or cell metabolism, and the authors favored a direct control of realizators by the Hox proteins instead of an indirect one through intermediate transcription factors.

As was also observed in *Drosophila*, the time of the analysis could greatly determine the downstream genes identified. For instance, in the work of Cobb and Duboule [59] the comparison of the transcriptional profile in forelimb and genitalia led the authors to conclude that many similar targets were regulated by the HoxD genes in the two structures, but at different times of development. In another example, the zebrafish *Hoxb1b* downstream genes identified at an early time of development, the gastrula stage [68], did not coincide with those recovered in a parallel study done with similar conditions and a paralogous Hox gene (*Hoxb1a*) [70], perhaps because both studies analyzed different developmental stages [70].

### 2.2. ChIP Experiments

As is the case for the microarray data, ChIP experiments with *Drosophila* Hox proteins have preferentially studied Ubx, analyzing the binding of the Ubx protein to the haltere disc chromatin [76–78]. One of the studies [76] found 1147 genes bound by Ubx, with a coincidence of 96% with those found in another study [77]. However, only 20% of the genes previously identified as downstream of *Ubx* in microarray or in situ hybridization analyses in haltere discs were detected in this ChIP experiment. The authors claim that if targets expressed at different times in haltere development were considered, as done in a previous microarray study [73], the figure could be higher. Out of the 1147 genes bound, 154 are associated with cellular functions like cell motility, cell adhesion (*shotgun*, *neuroglian*, and *cadherin-N*), cell communication, or apoptosis (*reaper*). Out of the 294 genes that were bound and validated, 69 of them (near 25%) belonged to one of these categories. The authors conclude that many genes bound by the Ubx protein are transcription factors or genes belonging to signaling pathways but that there is also a good representation of genes directly regulating basic cellular functions.

Another study [77] compared the genes bound by the Ubx protein in haltere and third leg discs, the two discs where *Ubx* is highly expressed. The authors found 3400 genes bound by Ubx in the haltere disc and 779 genes in the third leg disc. Out of the 488 genes previously identified as regulated by *Ubx* in expression profile experiments in the haltere disc [66], 191 were bound by *Ubx* in this ChIP experiment. This gives a higher correlation between the two types of experiments than in a previous ChIP analysis [76]. The targets include not only transcription factors (although they make up the majority class) but also genes involved in the Notch, Decapentaplegic (Dpp), and Wingless signaling pathways and genes regulating cellular processes such as cell cycle (*dacapo*, *cyclinE*, and *E2F*). This study also compared targets bound by Ubx in the haltere and leg discs, and also with those bound in the embryo, and found that 2705 genes bound in the haltere disc were not targets in the leg disc and that 84 bound in the leg disc were not targets in the haltere disc. The authors also found that, out of the 4590 binding peaks identified in the haltere disc, only 16% were also detected in the T3 leg disc and 42% in 0–12 h embryos (data provided from the modENCODE project; http://www.modencode.org/). These data indicate a high degree of specificity in binding by the Ubx protein depending on the tissue or organ. This conclusion is supported by the analysis of some genes, like *vestigial* or *cut*, with known function in dorsal but not ventral appendages: the authors found binding of Ubx to these two genes in the haltere (dorsal) disc but not in the leg (ventral) disc.

A third study [78] used different methods (Agilent platform, a different Ubx antibody) and found fewer genes [439] bound by the Ubx protein in the haltere disc (more precisely, wing pouch transformed to haltere pouch by a gain-of-function *Ubx* mutation) than the other two studies. However, the authors found many genes previously identified as being regulated by *Ubx*, such as *connectin*, *thickveins*, *spalt major*, and *scabrous*, thus validating their data. By analyzing the function of the genes bound by the Ubx protein the authors concluded that there were less number of genes corresponding to the realizator class (coding for cuticular, chitin-binding, cytoskeletal proteins, etc.) than those coding for transcription factors or forming part of signaling pathways. However, by including genes that are not exactly realizator genes but that determine cellular functions like cell proliferation, cell shape, and apoptosis, the number significantly increases. Comparison with targets obtained in two previous microarray studies [64, 73] showed that only around 5% [64] or 20% [73] of the genes coincide with those identified in this study. For example, out of 542 genes reported in Mohit et al., 2006 [64] as specifically regulated by Ubx in microarray experiments, only 26 are direct targets. This ChIP analysis, as the two previous ones, reveals that many of the genes bound by Ubx code for proteins involved in signaling pathways and this is in agreement with previous data showing the regulation by *Ubx* of many of these pathways in the haltere disc [64, 66, 91–102].

Finally, ChIP experiments with *Drosophila* Hox proteins were also carried out looking for genes bound by Dfd in stage 10–12 embryos [79]. The authors combined this experiment with an *in silico* identification of Dfd-specific Hox response elements. This was done by searching for regulatory sequences with Dfd binding motifs that were conserved in noncoding regions of 12 *Drosophila* species genomes. In this way, they identified 2012 Dfd enrichment peaks, including those belonging to previously characterized *Dfd* targets, like *reaper*. They also showed that Dfd and Ubx bind to different DNA sequences in stage of 10–12 embryos, suggesting a high specificity of binding *in vivo.*

In vertebrates, McCabe and Innis [74] used a genome-wide screen to identify genes bound by Hoxa13 in mouse embryonic fibroblasts. Among the targets identified they studied particularly *Enpp2*, a gene highly upregulated in these cells and required for cell motility. Another study [75] used ChIP-on-chip technology to identify genes bound by Hoxd13 in a cell line of mesenchymal derivation that stably expresses the Hoxd13 protein. The authors identified 248 genes, many of which belonged to the category of realizator genes (mainly genes involved in cell cycle, cell proliferation, cell adhesion, cytoskeleton, cell metabolism, and cell signaling). Many of the genes bound and analyzed form part of the regulatory network which determines the formation of the vertebrate limb, including genes such as *Bmp2* and *Bmp4*, *Hand2*, and *Dach1*, with some of them being bound only at specific times of development. Finally, another study [80] used ChIP-seq to identify genes bound by the Hoxa2 protein in the mouse second branchial arch, which is affected by inactivation of *Hoxa2*, at a precise time in the development (E11.5). The authors found that the Hoxa2 protein binds thousand of genes, with a preference for genes involved in the Wnt pathway.

### 2.3. General Conclusions of Genomic Approaches to Identify Hox Targets

Microarray and ChIP analyses have significantly contributed to identify Hox downstream genes, providing a more extensive, genome-wide, repertoire of Hox targets than that available from previous studies. Microarrays identify many genes differentially expressed but cannot distinguish between direct and indirect targets, although the former group predominates when the analysis is done just after the controlled induction of the Hox gene [67, 73]. On the other hand, ChIP identifies regions bound by Hox proteins, but that does not necessarily mean that the gene expression is regulated at the time of the analysis (or even that it is regulated at all). In fact, some of the targets bound by the Ubx protein at a certain developmental time point are expressed later on [49, 76]. Therefore, it is not surprising that there is no very high coincidence in the Hox targets identified by microarrays or ChIP data for the same Hox protein (Ubx) and in the same tissue (haltere disc) [64, 66, 73, 76–78]. In any case, the data obtained from the microarrays and ChIP data, together with previous studies, allow to draw some conclusions.

(1) Although many of the direct Hox targets code for transcription factors, a significant fraction of them are realizator genes. ChIP experiments in *Drosophila*, mouse, or cultured cells have identified many targets that code for proteins carrying out basic cellular functions [75–80], although in one of these studies the proportion is low [78]. Similarly, in those microarray experiments in which a high proportion of the genes identified are likely to be direct targets [67, 73] a significant number of these belong to the category of realizator genes. This suggests that Hox control of morphologies can be achieved, at least in part, without the need for the coordination of realizator genes through secondary transcription factors. However, the analysis of organ specification by Hox genes suggests otherwise (see below). It is possible that realizator genes are controlled both directly by Hox proteins and through intermediate transcription factors.

(2) Hox proteins govern genes involved in the same pathway in a similar fashion. In two microarrays studies [53, 67] the authors found evidence for coordination in the regulation of Hox targets: genes involved in a similar process, like apoptosis, cell adhesion, or cell cycle, are regulated together in the same direction (activation or repression).

(3) There is a correspondence between the morphological complexity, with respect to a homologous structure, elicited by a Hox protein, and the number of genes it regulates. The best example is the work of Slattery et al., 2011 [77]. The authors found genes bound by the Ubx protein in the haltere but not the leg disc and vice versa, but the number of genes specifically bound in the leg but not the haltere disc is much smaller than the other way around. This is not unexpected: *Ubx* differentiates T3 legs from T2 legs and halteres from wings (and the corresponding proximal regions), but the morphology of T3 and T2 legs is more similar than that of halteres and wings (this also applies to the domains of the two discs making the non-appendage regions in the adult). The higher morphological complexity of wings with respect to halteres correlates with the data obtained in two microarray studies: in one of them [66] it was found that 174 genes were more abundantly expressed in the wing disc than in the haltere disc but that only 18 genes were predominantly expressed in the haltere disc. In the other study [64], 16 genes were found to be more expressed in the wing disc than in the haltere disc and 7 genes the other way around. In agreement with this reasoning, another study found that just a few genes were differentially expressed in T1 and T2 leg discs (T1 leg is determined by *Scr*) [61], probably because the morphology and size of these two legs are similar. It was also discovered, however, that the same set of Hox proteins can form quite different organs, mouse limbs, and genitalia, through subtle modulation of the amount and temporal expression of a largely common group of targets [59].

(4) Most of the Hox targets are regulated at particular developmental stages [59, 67, 73]. Some genes are bound by Ubx in the third larval stage but respond to Ubx activity later on [49, 76], suggesting that binding and regulation may not always be coupled. In another example, ChIP analyses done at different developmental times identified different sets of genes [75]. A time difference may also explain why in two studies done with two paralogous Hox genes (*Hoxb1a* and *Hoxb1b*) there was no overlap between the identified targets [68, 70]. Time of activation of some Hox genes has been proved, in fact, to be essential for their function [103, 104].

(5) The number of genes similarly regulated by different Hox proteins has been found to be low [67], although common targets are identified when downstream genes from paralogous Hox genes are compared [65, 68, 72]. Different Hox proteins bind to different targets, as shown when comparing binding by the Dfd and Ubx proteins in the embryo, and the molecular architecture of the sequences that are bound by these Hox proteins is also different [79]. However, the different activity of Hox proteins could be due to some genes being specifically regulated by just one Hox protein or to subtle variations in the regulation of a common set of targets [59, 69]. Moreover, the existence of a certain number of common targets is supported by examples in which different Hox genes can perform the same role in development: *Ubx* and *abd-A* can similarly make gonads [105] or halteres [106], and several Hox genes can make the tritocerebrum [107]. Although in some of these experiments the potential similar role of Hox proteins, rather than their actual function in development, is compared, these analyses, and the similar morphology observed among segments with different Hox expression in *Drosophila*, suggest that in some cases different Hox genes may regulate common targets.

(6) The regulation of Hox targets can be highly influenced by the presence of cofactors or collaborators, which help in Hox protein binding and target regulation [23, 102, 108–110]. This may explain why a particular Hox protein regulates some targets at a precise developmental stage and in a restricted number of cells.

A strict hierarchical architecture whereby Hox proteins regulate cellular functions through intermediate genes is probably not a general rule, although in some cases Hox genes seem to follow this regulatory structure ([111]; see below). Nevertheless, even in this case dual regulation, direct and indirect, of some realizator genes, cannot be excluded. The regulation of Hox targets in different tissues and times of development, in collaboration with many different proteins [23, 47, 79, 102, 108–110], suggested that Hox proteins could act more as “micromanagers” than as selector genes at the top of a well-established hierarchy [112]. Whatever the way they act, Hox genes must regulate many downstream targets to control cellular behavior.

## 3. The Cellular Functions

Since Hox mutations frequently cause major changes in morphology and Hox proteins are also sufficient to develop new organs, it was expected that Hox genes should modify many cellular processes needed for those changes. I describe in what follows some examples of cellular functions regulated by Hox genes.

### 3.1. Cell Death 

Apoptosis, or programmed cell death, is an important mechanism in the development of some organs [113]. Hox genes regulate apoptosis in different tissues and organisms. One of the best-characterized examples involves the regulation of cell death by the Hox gene *Dfd* in the *Drosophila* embryo [114]. *Dfd* is required for the development of the mandibular and maxillary segments, two lobes in the posterior part of the head separated by a groove in the epidermis [115, 116]. Two characteristics of *Dfd* mutant embryos are that they show an excess of cells in the ventral part of these two segments and that the groove that separates them is eliminated. Expression of the proapoptotic gene *reaper* (*rpr*) and subsequent cell death are observed in the cells that form this boundary in wildtype embryos, and embryos homozygous for a deficiency uncovering three proapoptotic genes, *rpr*, *head involution defective* (*hid*), and *grim*, present a phenotype (absence of the groove) similar to that of *Dfd* mutants. In *Dfd* mutants *rpr* expression (and cell death) is eliminated, and *Dfd* is sufficient to activate *rpr* when expressed ectopically in the embryo. It was further demonstrated that *Dfd* binds to the *rpr* regulatory region, that this binding is required to activate *rpr*, and that the expression of *rpr* is sufficient to make the boundary even in the absence of *Dfd* [114]. This example demonstrates that cell death is a cellular process required for morphogenesis directly regulated by a Hox gene.

A similar control of cell death and morphogenesis was reported for another Hox gene, *Abdominal-B* (*Abd-B*). *Abd-B* is needed to determine the posterior abdominal segments (A5 to A9) [6, 7]. In mutants for this gene the segment boundaries between the sixth and seventh, or the seventh and eighth, embryonic abdominal segments are partially suppressed, so that a weak fusion between adjacent segments is observed. There is *rpr* transcription in these boundaries, and this depends on *Abd-B*. Moreover, *Abd-B* is able to induce *rpr* expression ectopically. Therefore, both *Dfd* and *Abd-B* are able to activate *rpr* and cell death, and this activation impinges in morphological changes on the embryo [114]. However, the regulation of cell death by *Abd-B* is implemented in a dual manner: as I have just described, *Abd-B* regulates *rpr* expression and development of the grooves between segments [114]; however, in these same mutants there is ectopic *rpr* expression at the boundary between the A8 and A9 segments in stage 12 and later embryos [117].

Apart from *Dfd* and *Abd-B*, other Hox genes control *rpr* expression in *Drosophila* embryos. *lab* is required for the development of the procephalic lobe [83] and in stage 12 *lab* mutant embryos *rpr* expression is increased in this lobe, indicating that *lab* represses *rpr* in these cells [117]. Similarly, *Scr* and *Antp*, two genes of the ANT-C, repress *rpr* in stage 13 or 12 embryos, respectively, as detected by the ectopic expression of *rpr* observed in posterior head or thoracic segments, respectively, in *Scr* or *Antp* mutants [117].

Well-characterized examples of the regulation of cell death by Hox proteins occur in the *Drosophila* embryonic nervous system. In this tissue neuroblasts divide as stem cells, each of them giving rise to another neuroblast and to a smaller cell, the ganglion mother cell, which normally divides only once to give neurons or glia [118, 119]. At postembryonic stages, neuroblasts continue dividing but there are differences in their development along the anteroposterior axis [120]. In the larval ventral cord, a rise in the levels of the Hox protein Abdominal-A occurring in the abdominal segments during the second larval period causes apoptosis through the activation of one, two, or three of the proapoptotic genes, *rpr*, *hid*, and *grim* [121]. Therefore, *abd-A*, by controlling apoptosis when the larva enters the third larval stage, limits the number of progeny produced by neural precursors in the larval abdomen [121–123].

The survival of some neurons at the back of the embryonic ventral cord depends on *Abd-B* [124]. The dMP2 and MP1 pioneer neurons, which are generated along the ventral nerve cord, undergo apoptosis, after their early pioneering function, only in anterior segments. *Abd-B* prevents the death of the post mitotic neurons dMP2 and MP1 by repressing *rpr* and *grim*, and if *Abd-B* is expressed anteriorly it rescues cell death of more anterior neurons [124]. Another example of Hox-regulated cell death in this tissue is provided by *lab*. This Hox gene is required in two specific neuroblast lineages of the brain to induce cell death. In *lab* mutants, there is no cell death and two ectopic neuroblast lineages are formed [125].

The effect of the *Abd-B* gene in preventing cell death contrasts with its apoptotic-promoting role not only in the ectoderm [114] but also in some other neurons [126]. In the larval VNC, six peptidergic neurons (capability expressing Va neurons) express the Capa neuropeptides, encoded by the capability gene [127]. Generated initially in all the segments, the neurons present in the A5–A8 segments die: here the role of *Abd-B* is proapoptotic and mediated by the same proapoptotic genes repressed by *Abd-B* in the dMP2 and MP1 neurons [126]. Similarly, one of the two proteins encoded by the *Abd-B* gene, AbdBR, triggers programmed cell death of several progeny cells within the NB3-3 lineage of the embryonic ventral cord [128]. Another proapoptotic gene, *sickle*, is strongly expressed at the back of the ventral nerve cord at late stages of embryogenesis, also suggesting a positive regulation of cell death by *Abd-B* [117].

The Hox genes *Ubx* and *Antp *provide further examples of Hox genes controlling cell death in the embryonic nervous system. *Ubx* is upregulated in motoneurons of the central nervous system (CNS) at late embryonic stages, and *Ubx* activates *rpr* and induces cell death in these neurons [129]. Contrary to this, *Antp* is required for these cells to survive, and in the presence of the two products *Ubx* function prevails and the cells die [129]. Finally, recent studies have shown that the absence of *Antp* (and *Ubx*) promotes cell death in leg motoneurons [130]. As a summary, these studies not only reveal that Hox genes have a major role in regulating cell death in the *Drosophila* nervous system but also provide evidence of the importance of the context in regulating Hox activity since the same proteins, Ubx or Abd-B, can be proapoptotic or antiapoptotic in the same tissue.

Apart from *Drosophila*, there have also been reports of Hox genes governing cell death. For example, *Hoxb13* both induces cell death and represses proliferation in the caudal spinal cord of mice [131]. By contrast, inactivation of *Hoxc8* causes an increase of apoptosis in cervical and thoracic (C7-T1) motoneurons in mice [132], and in the absence of *Hoxb1 *multiple neurons normally specified within rhombomere r4 are instead programmed for early cell death [133]. Outside the nervous system, one of the most studied examples of programmed cell death is the need of apoptosis to separate digits in the vertebrate limb [134]. Mice lacking the *Hoxa13* gene show reduced levels of cell death and syndactyly [135, 136]. The effect of *Hoxa13* on cell death is mediated, at least in part, not only by the regulation of *Bmp2* and *Bmp7* [136], but also by the control of retinoic acid production, a key step in controlling cell death in the limb [134]. *Aldh1a2*, the gene encoding the enzyme that converts retinaldehyde to retinoic acid and so regulates interdigital programmed cell death by controlling retinoic acid signaling, is directly regulated by *Hoxa13* [137]. *Hoxa13*, therefore, maintains interdigital cell death through the regulation of Bmps and retinoic acid [137].

In *C. elegans*, the Hox gene *lin-39* (orthologous to *Drosophila Scr*) is necessary for the survival of six neurons of the central region of the ventral nerve cord [138] through the repression of the BH3 only cell death gene *egl-1* [139, 140]. By contrast, *mab-5* (orthologous to *Drosophila Antp*) is needed for the programmed cell death of two neurons generated in the P11 and P12 lineages P(11, 12).aaap cells [141, 142].

### 3.2. Cell Migration

The best examples of the control of cell migration by Hox genes are found in the nematode *C. elegans*. In the worm, two neuroblasts, QL and QR, derive from the division of the Q neuroblasts and are located in the posterior part of the embryo. The neuroblast QL and its descendants migrate posteriorly in the wildtype, but in mutants in the Hox gene *mab-5*, they instead migrate anteriorly. A gain-of-function allele of *mab-5* makes QL and QR descendants migrate posteriorly, indicating that *mab-5* is necessary and sufficient for migration [143, 144]. Similarly, the QR neuroblast and its descendants migrate anteriorly in the wildtype but in mutants for *lin-39* this migration does not occur [138, 145].

Transcriptome studies were used to identify *C. elegans* Hox targets involved in migration. RNAseq analysis of *wildtype*, *mab-5* loss-of-function and *mab-5* gain-of-function mutants identified, among the genes putatively regulated by this Hox gene, an enrichment in genes encoding transmembrane and secreted proteins, particularly those involved in cellular matrix formation and rearrangement, but not many genes encoding transcription factors. About one third of the genes identified as being upregulated in *mab-5* gain-of-function alleles and downregulated in *mab-5* loss-of-function mutants had an effect in the migration of Q descendant when inactivated with RNAi [146]. One of the genes required for the anterior migration of neuroblasts is *mig-13*, encoding a transmembrane protein [147]. The effect of *lin-39* in the anterior migration of QR descendants is mediated by its direct control of *mig-13*, which regulates actin accumulation in the leading edge of the cells that migrate; *mab-5 *and Wnt signaling, by contrast, repress *mig-13* expression to ensure posterior migration of QL descendants [148].

### 3.3. Cell Affinities

Old experiments in *Drosophila* imaginal discs suggested a role for Hox genes in determining different affinities. Cells from different imaginal discs do not intermingle when put together in culture [149–151] and in some cases this is due to the activity of Hox genes. For example, wing and haltere cells sort out in cultured cells but wing cells mix with haltere cells mutant for *bithorax* or *postbithorax* mutations, which reduce *Ubx* expression in the haltere disc thus transforming it into a wing disc [151].

Other experiments also argue for an important role of Hox genes in establishing different cell affinities. In *Drosophila*, clones mutant for *Ubx*, *Abd-B*, or *Dfd* sort out from the rest of the tissue [88, 152–156]. In the eye-antennal disc of *Drosophila, Dfd* segregates cells of the maxillary primordium from those in the rest of the disc, establishing a clonal boundary between cells expressing and not expressing *Dfd* [154]. The idea that a different Hox activity can maintain cell lineage restrictions is supported by other experiments. The wing, haltere, and leg discs are subdivided into anterior and posterior compartments from the embryonic stage, and cells from either compartment do not mix with cells from the other one throughout development [157, 158]. Higher expression of myosin II at the boundary between both compartments may help to segregate anterior and posterior cells through local increase in mechanical tension [159, 160]. It has been reported that differences in *Ubx* expression can also increase the levels of myosin at this boundary and maintain segregation of cells from different compartments [156]. The role of Hox genes in regulating cell affinities may be mediated also by cadherins. In the posterior spiracles of the embryo, *Abd-B* regulates the expression of cadherins, which are probably needed to maintain cohesion of the tissue during the morphogenetic movements that results in spiracle development [111, 161].

Targets of vertebrate Hox genes also include cell adhesion molecules. In fact, the first Hox target identified in vertebrates was the mouse neural cell adhesion molecule (N-CAM), which is needed for cell adhesion in the nervous system [162]. In the vertebrate hindbrain there is segregation of populations of cells from adjacent rhombomeres [163], and these structures are characterized by their unique expression of Hox genes [90, 164, 165]. Hox genes maintain the normal development of rhombomeres [166] perhaps by providing the tension required to maintain segregation of cells through the control of actomyosin molecules [167]. Therefore, Hox genes help to segregate tissues with different fate and prevent mixing of cells that differentiate into particular structures perhaps through the control of actomyosin and cadherin activity.

### 3.4. Cell Proliferation

Several examples of the connection between Hox genes and cell proliferation have been reported. For instance, *Hoxa10* regulates *p21*, coding for a cyclin-dependent kinase inhibitor, in differentiating myelomonocytic cells [168]. In Rat-1 cells, *Hoxb4* induces cell proliferation through the control of AP-1 activity, which in turn regulates cyclin D1 [169], and in breast cancer cell lines *Hoxa5* stimulates the transcription of the tumor-suppressing gene *p53* [170] (reviewed in [171]). In *Drosophila*, *Abd-B* regulates directly the expression of *dacapo*, which codes for an inhibitor of CyclinE/Cdk2 complexes, in the *Drosophila* embryonic epidermis [172]. Within the *Drosophila* embryonic nervous system, *Ubx* and *abd-A* regulate proliferation of neuroblasts, controlling the number of cell divisions they undergo during larval stages [173], and in the *Drosophila* postembryonic brain *lab* is required in two neuroblast lineages to end cell division by activating programmed cell death [125]. In *C. elegans*, *lin-39* is necessary for all cell divisions that occur in the vulva after Ras activation [174].

Homeotic mutations convert all the characteristics of one organ into those of another one; among them is its size. This is most evident in some of the more conspicuous *Antennapedia* or BX-C mutations. For example, the ectopic expression of the *Antennapedia* gene in the antennal primordium transforms antennae into legs [32–34]. Antennae are much smaller than legs, and so this transformation involves a change in organ size probably due to changes in cell proliferation. Hox genes, therefore, are likely to regulate the size of different organs by directing changes in cell proliferation [175].

The regulation of growth by Hox genes has been studied in more detail in the control of wing and haltere size by the gene *Ubx*. Wing discs are about 4-5 times the size of haltere discs at the end of the third larval period, a difference due to *Ubx* expression [97, 98]. Small *Ubx* mutant clones induced in the haltere disc, however, grow as the rest of the cells of the disc, indicating that *Ubx* does not control cell proliferation rate autonomously. However, big *Ubx* clones can overgrow with respect to surrounding cells, and this is due to the effect of *Ubx* mutant cells on the expression and activity of the growth factor *dpp* [97, 98, 101]. The posterior compartment of wing and haltere discs secretes the Hedgehog protein into the anterior cells, activating *dpp* expression at the anteroposterior compartment boundary. *dpp* encodes a protein of the BMP2, 4 family that is required for growth in imaginal discs [176, 177]. The Dpp protein, synthesized at the anteroposterior compartment boundary, spreads from this position to both anterior and posterior compartments promoting growth in both of them [177, 178].

The *dpp* band of expression is wider in the wing disc than in the haltere disc, and in mutations that reduce *Ubx* expression in the anterior compartment of the haltere disc the *dpp* band extends anteriorly. *Ubx*, therefore, reduces *dpp* expression. In addition, *Ubx* regulates the spread of Dpp and therefore Dpp activity in the whole disc. This is done through the control of the levels of expression of the main type I receptor of the Dpp ligand, encoded by the gene *thick veins*, and through the control of *dally* and *dally-like*, two genes coding for proteins that contribute to the spread of Dpp [179–184]. *Ubx*, by increasing *thick veins* expression in the haltere disc, limits the spread of Dpp, since a substantial amount of ligand molecules are bound to the increased number of receptors, thus reducing Dpp signaling in cells away from the source. Besides, by diminishing *dally* and *dally-like* expression, *Ubx* similarly reduces Dpp diffusion. The end result of these regulations is that *Ubx* reduces *dpp* expression and Dpp spread. In *Ubx* mutations, both expression and spread increase, leading to more cell proliferation and to an increase in haltere disc size comparable to that of the wing disc. The effects on cell proliferation and on cell size (described below) lead to a complete transformation of haltere size into wing size in *Ubx* mutants [97, 98, 100, 101].

Neuroblasts proliferate in the *Drosophila* embryo and give rise to a wide range of neurons. After embryogenesis, most neuroblasts of the abdomen die by apoptosis, but most of those in the cephalic or thoracic regions enter into quiescence and reenter mitosis during the larval period. One example of Hox control of neuroblast entry into quiescence has been described with the NB3-3 neuroblasts. *Antp* is expressed in the NB3-3 thoracic neuroblasts and *abd-A* in the abdominal ones. In *Antp* mutants, or after ectopic *abd-A* expression, some of these thoracic neuroblasts divide at the end of embryonic development instead of entering quiescence. Thus, *Antp* and *abd-A* spatially restrict the entry of NB3-3 neuroblasts into quiescence [185].

Outside *Drosophila*, some experiments also connect Hox gene activity and cell proliferation. For example, the *mab-5* and *lin-39* Hox genes in *C. elegans* stimulate cell proliferation in certain cell lineages. Lateral ectoderm cells (V cells) require *mab-5* for proliferation in the L2 stage [186] and in the formation of the vulva it was found that *lin-39* is needed to activate a gene required for cell division [187].

### 3.5. Cell Size

One good example of how Hox genes regulate cell size is provided by the control by *Ubx* of cell size differences between wing and haltere. Wing cells are much bigger than haltere cells, and this difference occurs during the pupal stage. During this period, wing cells greatly expand to acquire a much bigger size than those in the haltere. In the pupal haltere disc, *Ubx* represses this expansion, thus contributing to the great size differences between wings and halteres in the adult [188]. The genes regulated by *Ubx* to implement this size difference are unknown.

## 4. Organogenesis

Hox genes may promote new morphologies in at least two major ways [43]: (a) Hox genes can modify a well-defined basic pattern which provides the genetic information defining the general layout of a certain structure, or (b) Hox genes can promote the development of new structures with a reduced genetic layout. This separation into two classes, however, is not always strict, since the extent of the basic general pattern modified by Hox genes varies with each structure. In the first group are included modifications of serially homologous structures, such as the development of the haltere instead of the wing in *Drosophila* or the formation of abdominal vertebrae instead of thoracic ones in the mouse. In these cases, Hox genes just modify, although sometimes very significantly, a structure with a well-defined underlying plan. The most famous homeotic mutations, such as the antenna to leg transformation or that resulting in the four-winged fly, are among the best examples of changes in homologous structures due to the different activity of Hox genes. These changes are not simple modifications of the final differentiation state. As I have described for the haltere, *Ubx* changes the outcome of different signaling pathways and in this way significantly modifies size and pattern. However, even in those cases the basic developmental plan of the haltere and wing disc is very similar, and halteres can just be considered as modified wings, not new structures.

A second class of Hox-mediated organogenesis is provided by the formation of new organs without homology in the rest of the animal. Examples within this group are the formation of salivary glands or posterior spiracles in *Drosophila*. In this second class, Hox genes do not modify a previously established organ plan but promote the development of a new structure with a reduced background patterning information (defining dorsoventral, anteroposterior, and compartment cues), that is, without a clear homology to any other structure in any other part of the body. Mutations that affect this Hox activity do not give rise, in general, to any recognizable transformation of pattern; thus, mutations in the *Drosophila* Hox genes *Dfd* [115, 116] or *lab* [83] show abnormal or absent structures, but no major homeotic transformations. In some instances, mutations in a single Hox gene can give rise to recognizable transformations or absence of a structure depending on the tissue or organ considered. In any case, Hox genes must coordinate cellular functions to obtain a harmonious development of organs. I will discuss organogenesis in different classes of Hox activity.

### 4.1. “Classical” Hox Activity

**Figure 1:**
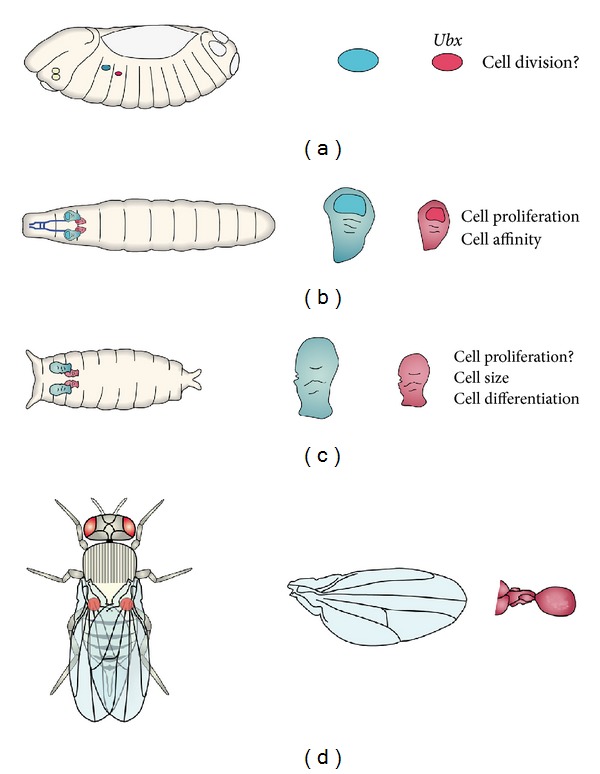
Different processes that lead to the development of halteres instead of wings by *Ubx*. Halteres and haltere discs are in red, whereas wings and wing discs are in blue. (a) At the end of the embryonic development, Ubx activity makes the haltere disc about half the size of the wing disc. (b) During larval development, *Ubx* regulates many signaling pathways to determine haltere disc size and to specify haltere development. (c) During pupation, *Ubx* reduces cell size in the haltere disc as compared to that of the wing disc and also determines cell differentiation. The final result is the development of a haltere instead of a wing (d).

*(a) Development of the Drosophila Halteres. Ubx* regulates the formation of halteres instead of wings by coordinating several processes during embryonic, larval, and pupal development. At the end of embryogenesis, haltere discs are about half the size of wing discs due to the early action of *Ubx* in disc size [189, 190]. During larval development, *Ubx* regulates the activity of different signaling pathways (*dpp*, *wg*, *EGFR*, etc.) at different levels within the pathway [64, 66, 91–102]. The regulation of these pathways controls size and pattern, thus promoting haltere development. During pupal stages, *Ubx* induces substantial changes in cell size and cell differentiation, leading to the final development of the halteres as opposed to wings [73, 188] (Figure 1).

*(b) Development of the Drosophila Legs.* In contrast to the major differences observed between wings and halteres, the three legs of *Drosophila* differ just in a few characteristics relating to size and pattern, mainly in bristle arrangement and in the presence of the sex comb in the T1 male leg [191, 192]. The first leg differentiates from the second one by the expression of the Hox gene *Scr* and the third leg acquires its identity by the activity of *Ubx*. Accordingly, mutations in *Scr* transform the T1 leg into the T2 one [84, 85, 193] and *Ubx* mutations the T3 leg into the T2 one [85, 194–197] (there is also an early function of *Ubx* to differentiate T2p and T3p from T1p; [103, 195]).

These transformations involve just relatively small changes in leg size or in the spatial arrangements of bristles [192–201]. The latter is achieved through the precise control of genes determining bristle development. For instance, in the distal part of the T2 leg there is restricted expression of the genes *achaete* and *scute*, responsible for the formation of the bristles, due to the combined activity of the Hairy repressor and the Notch ligand Delta [198, 199]. *Scr* and *Ubx* determine the specific pattern of bristles in the T1 and T3 legs, respectively, by regulating *Delta*, which, in turn, controls *achaete* expression and bristle development [200]. *Ubx* also regulates the formation of large macrochetae in T3 legs, at different times of development [201]. These studies highlight the fact that the general control of size and pattern is similar in the three legs (but with some significant differences) and that the major role of Hox genes is to determine changes in bristle pattern at late developmental stages. The absence of major size differences between legs suggests that changes in, for instance, the Dpp signaling pathway, which differentiate haltere and wing disc size, do not operate, or do it much more weakly, in the metathoracic leg discs (ventral T3) than in the haltere disc (dorsal T3), suggesting a different activity of *Ubx* on Dpp activity ventrally and dorsally within the same segment. As I have explained above, the relatively small differences between legs correlate with the small number of Hox targets that change gene expression among leg discs [54, 61] and with the small number of genes bound by *Ubx *exclusively in the third leg disc as compared to the haltere disc [77].

*(c) Suppression of Structures. *An extreme case of modification of one structure by a Hox gene is when such a structure is eliminated. In these examples, mutations in Hox genes prevent the suppression of the structure by promoting the formation of an organ characteristic of a different part of the body.

One example of such Hox activity is the suppression of legs in *Drosophila*. In the fruit fly, development of the legs requires the expression of the gene *Distal-less* (*Dll*; [202, 203]). *Dll* homologues are also required to specify limbs both in protostomes and deuterostomes [204, 205]. *Dll* is expressed in the embryonic leg discs, located in the thoracic region [206–208], whereas in the abdomen *Dll* expression is directly prevented by the Hox genes *Ubx*, *abd-A*, and *Abd-B*, and in mutants for these genes there is ectopic *Dll* expression in the abdominal region [109, 209–212]. These studies show that *Dll* is an example of a common Hox target, since it is repressed by three Hox genes, *Ubx*, *abd-A*, and *Abd-B*.

Another example of suppression of a structure mediated by a Hox gene has been described in the abdomen of the fruit fly. In *Drosophila*, females have seven abdominal segments (although the seventh one is smaller than the others) but males lack this segment. The elimination of this metamere depends on the sex determination pathway and on the activity of the Hox gene *Abd-B*: in mutants that convert males into females or in *Abd-B* mutants, a seventh segment develops in the male abdomen [6, 7, 213–215]. The abdominal segments derive from histoblasts, cells that are grouped in discrete clusters called histoblast nests and that do not proliferate during the larval period. These clusters are surrounded by large, polytenic larval cells that during pupal stages are extruded, die, and are replaced by the dividing and expanding histoblast nests [189, 216, 217]. The nests of the male seventh abdominal segment do not differ in any respect from those of more anterior segments or from the female ones until pupation [216]. However, during pupation these histoblasts show a lower proliferation rate than that of the other histoblast nests, are finally extruded, and die [218, 219]. Some genes and pathways are differentially active in the histoblasts of the male A7 and in more anterior segments during pupal stages, and these genes are regulated by *Abd-B*. Thus, *wg*, a Wnt ligand, is expressed in the dorsal male histoblasts except in the A7; similarly, the EGFR pathway is less active in this same segment [218, 219] and the gene *extramacrochetae*, coding for a HLH protein [220, 221], is also required for the suppression of this segment [219]. Since *Abd-B* also modulates the expression of *doublesex*, the gene at the end of the sex determination cascade [219, 222], the cellular functions (cell proliferation, cell extrusion, and cell death) that result in segment elimination ultimately depend on the activity of *Abd-B* in the male A7.

### 4.2. Formation of New Organs

Hox genes must coordinate different cellular processes to build structures. The complex processes required for this task are being dissected using relatively simple organs where Hox activity could be more easily analyzed. Two examples of this approach are the studies of development of posterior spiracles and of salivary glands, both in *Drosophila*.

#### 4.2.1. Posterior Spiracles

The posterior spiracles (PS) are structures derived from the ectoderm that protrude at the back of the *Drosophila* embryo. They are needed to connect the respiratory system (the longitudinal tracheae) with the outside and are composed of two parts: the spiracular chamber, which makes contact with the trachea, and the stigmatophore, the part that protrudes. Within the spiracular chamber there is a refractile filter called the filzkörper. PS develop in the embryonic eighth abdominal segment (A8) during 6–13 h of development and their formation requires the coordination of several cellular processes including changes in cell shape, cell rearrangements, cell adhesion, and cell polarity. The combination of simple organization and relatively complex cellular mechanisms makes the PS a tractable model to study the basis of Hox-directed organ formation [111, 223].

At stage 11 of embryonic development some 80–100 epidermal cells at the dorsal part of the A8 undergo apical constriction and invaginate in a sequential order: the anterior cells invaginate first and connect with the trachea, whereas the more posterior cells invaginate later on, developing the more external region of the spiracular chamber. As this chamber is formed, the cells located at the outside, marked by the expression of the gene *spalt* (*sal*), surround those making the spiracular chamber, undergo extensive rearrangements, and develop the stigmatophore. During these processes there is no cell death or cell division [111, 223].

*Abd-B* is the Hox gene expressed in the posterior abdominal segments of the embryo and it is necessary and sufficient for the formation of the PS [6, 7, 215, 224–227]. Although *Abd-B* is expressed in the whole A8 [228, 229], PS develop only in the anterior part of the segment and in a dorsal position, and the PS induced after ectopic *Abd-B* expression also develop in similar locations in more anterior metameres. The position where they develop along the dorsoventral axis depends on the activity of the Dpp pathway, and if the activity of this pathway is provided uniformly, genes normally expressed in the PS, and therefore in a dorsal position, are present now in a dorsoventral stripe [111]. Therefore, spatial cues provided by genes that subdivide anteroposterior and dorsoventral axis constrict the activity of *Abd-B* in forming PS.

As explained above, the activity of Hox genes is frequently modulated by the presence of cofactors, such as those encoded by the genes *exd* and *hth*. In the *Drosophila* embryo, *hth* and *exd* expression (and Exd translocation to the nucleus) is repressed in the *Abd-B* domain [212, 230–232]. This repression is needed for posterior spiracle development, since forcing *hth* an *exd* expression at the back of the embryo impedes *Abd-B* activity and therefore this development [232]. *Abd-B*, then, regulates all the cellular processes needed for the formation of these structures in the absence of any of the well-known cofactors, although it may use other, still poorly characterized cofactors, like that encoded by the gene *lines* [233]. 

*(1) Primary Targets.* The activity of *AbdB* in forming PS is mediated through “intermediate” transcription factors or signaling pathways that carry out different cellular functions. These primary targets are *empty spiracles* (*ems*), *cut* (*ct*), *unpaired*, the ligand of the JAK-STAT pathway, and *sal* [161]. *ems*, *ct*, and *up* are expressed in precursors of the spiracular chamber, in partially overlapping patterns, and *sal* in the cells giving rise to the stigmatophore, with a pattern complementary to that of *ct* [161, 223, 234]. The subdivision in two groups of cells developing different structures (spiracular chamber and stigmatophore) and with a complementary expression of two genes, *ct* and *sal*, suggests that *Abd-B* constructs spiracles by the subdivision of tasks. Each of the four primary targets fulfills specific functions in PS development.

*Spalt (sal). sal* encodes a zinc finger protein [235] and is expressed in the cells forming the stigmatophore. *Abd-B* activates *sal* and *sal* activates the gene *grain*, which finally determines the rearrangements of the stigmatophore [236]. Mutants in *sal* form spiracular chambers but no stigmatophore [111].

*Empty Spiracles *(*ems*). *ems* is expressed in PS and is directly, activated by *Abd-B* [237]. In *ems* mutants the cells do not invaginate; there is no filzkörper and no connection of the trachea with cells at the surface [238]. The spiracular chamber, therefore, does not form properly in these mutants, but the absence of *ems* does not affect elongation of the cells of the spiracular chamber [161].

*Cut.* The gene *cut* codes for a homeodomain-containing transcription factor [239]. In *ct* mutants the spiracular chamber is incomplete: the trachea connects to the spiracle but there is no filzkörper [111]. *ct* controls differentiation of the filzkörper and prevents apoptosis by directly regulating the proapoptotic gene *rpr* [240]. Like *ems* mutations, *ct* mutations do not affect the elongation of the spiracular chamber [161].

*Unpaired 1 *(*upd*) and* Unpaired 2.* These two genes code for two ligands of the JAK-STAT pathway [241, 242] and are expressed in the precursor cells of the spiracles under the control of *Abd-B* [161, 242]. In mutants that disrupt the JAK-STAT pathway there is no elongation of the cells of the spiracle, and the cells of the spiracular chamber remain at the surface displacing the stigmatophore [161].

Mutations of the four genes completely eliminate spiracles and, importantly, simultaneous expression of the primary targets, *ems*, *ct*, *upd*, and *grain* (a gene downstream *sal*), in anterior regions of the embryo, forms ectopic spiracles even in the absence of *Abd-B* [161]. This indicates that they are the only *Abd-B* primary targets needed to develop the PS.

*(2) Cellular Processes and Secondary Targets.* The activity of primary targets regulates other genes (secondary targets) that implement different cellular functions (cell adhesion, cell polarity, etc.) to construct the organ.

*Cell Adhesion, Cadherins, and Invagination.* Cadherins are calcium-dependent adhesion proteins. Their structure is that of transmembrane proteins with an intracellular domain that can bind beta-catenin (classical cadherins) or not (nonclassical cadherins) [243]. One of the classical cadherins, *E-cadherin* (*E-cadh*), is expressed in the whole ectoderm but at higher levels in the presumptive spiracle cells, and four nonclassical cadherins are also expressed in different groups of cells of the spiracle: *cad88C* and *cad96C* are present in elongated cells that are deeply invaginated, *cad74A* is expressed in distal cells of the spiracular chamber, and cells from the stigmatophore express *cad86C* [161]. The expression of these cadherins (or their higher levels) depends on *Abd-B*, although indirectly: the expression of *cad88C* and *cad96C* depends mainly on *ems* and also on the JAK/STAT pathway, that of *E-cadh* on *ct* and and the JAK-STAT pathway, the expression of *cad74A *depends on *ct*, and that of *cad86C* depends on *sal*. The result of the complex regulation of these genes is a mosaic-type distribution of cadherins in the cells that develop the PS [161].

The effect of inactivating cadherin function was studied by injecting cadherin dsRNA in embryos. Reducing one or two non-classic cadherins simultaneously with this method does not produce any major effect in PS development. However, reducing both *cad88C* and *cad96C* in an embryo also mutant for *E-cadh* duplicates the frequency of PS cells that do not invaginate as compared to embryos just mutant for *E-cadh* [161].

*Regulation of the Cytoskeleton.* The Rho family of small GTPases controls different cellular processes, such as cell migration or cell rearrangements. They do so by reorganizing the cytoskeleton through changes in the distribution of actin and tubulin. They are activated by guanine nucleotide exchange factors (GEFs), which catalyze the exchange of GDP for GTP, and inactivated by GTPase activating proteins (GAPs), which control the ability of the GTPase to hydrolyze GTP to GDP, thus turning it to the inactive conformation [244]. There is localized distribution of GEFs and GAPs in cells of the PS. Thus, RhoGEF64C is located in the apical domain of PS cells and is activated by *Abd-B* through *ems* and the JAK-STAT pathway [161], RhoGEF2 accumulates also in the apical side, and the RhoGAP Crossveinless-c (Cv-c) localizes to the basolateral side of spiracle cells, in a complementary pattern to that of the two GEFs. *RhoGEF2 *and *cv-c* are also regulated by *Abd-B* [161, 245, 246]. It has been proposed that Rho1 is GTP-bound apically (where RhoGEF2 and RhoGEF64C are located) and GDP-bound on the basolateral side (where Cv-c is present) and, in this way, apicobasal polarity can be coupled to the control of the small GTPase Rho function during apical constriction and invagination of the PS cells [246].

The PS do not invaginate, or the spiracular chamber is suppressed, when the activity of these GTPases is down- or upregulated [161, 245, 246]. Similarly, the expression of dominant negative or constitutively active forms of Rho1 prevents normal spiracle invagination [161, 246], and embryos mutant for loss- or gain-of function alleles of *cv-c* show, in a certain proportion of embryos, defects in invagination of the PS and abnormal filzkörper [161, 245, 246]. These experiments demonstrate that the activity of GEFs and GAPs has to be tightly regulated to allow a normal development of the PS.

*Control of Cell Polarity.* Morphogenesis of the PS involves changes in the shape of cells and in the relative amount of basal or apical membrane surface. Elongating cells of the spiracular chamber undergo an expansion of the basolateral membrane, as shown by looking at the expression of apical proteins like Crumbs (Crb) or Echinoid. Crb is an apical transmembrane protein required to maintain apical polarity [247, 248] and accumulates in a subapical region. Crb must be correctly expressed and localized for the maintenance of the epithelium because in *crb* mutants the epithelium collapses [249]. The expression of *crb* is upregulated in the cells forming PS, and this increased expression, directed by a specific enhancer, is important for cell elongation [161, 250]. The higher levels of *crb* may help to maintain apical membrane, while laterobasal membrane is elongating, although it may also have a role in the localization of proteins to the apical membrane [111]. In *crb* mutants the PS are abnormally formed; the phenotype resembles that of mutants in the JAK-STAT pathway, and in fact *crb* expression is regulated by STAT [161].

*Coordination of the Regulation of Different Processes by Abd-B.* The different processes taking part in PS development require the activity of secondary genes, as those just described, and the coordination between different cellular activities. Several other genes have been shown to be expressed in the PS primordium and controlled by *Abd-B* and some of the primary targets [117]. These include *arrowhead*, coding for a LIM-homeodomain transcription factor [251], *quail*, coding for a villin-like, actin binding protein [252], *pox neuro*, encoding a paired-box transcription factor required to determine the poly-innervated sense organs in *Drosophila* [253, 254], *senseless*, encoding a Zn-finger transcription factor required in the peripheral nervous system [255], and *shifted*, coding for the *Drosophila* ortholog of the Wnt inhibitory factor-1, which controls the distribution of Hedgehog in the wing disc [256, 257]. How the proteins coded by these genes integrate in the cellular processes described within the PS is unknown.

A recent publication reports on the coordination of gene activity required to produce PS [250]. *Abd-B* increases the expression of some genes, with otherwise ubiquitous expression, in cells forming the PS. One of these genes is *crb*, whose increased expression is necessary for the elongation of invaginating cells in the PS. Such increase is deleterious to cells outside this structure, but in the PS it is compensated by a concomitant rise in the amount of other proteins like RhoGEF64C, E-Cadh, or aPKC. Therefore, *Abd-B* increases *cv-c* expression to help invagination of spiracles but, at the same time, increases the levels of other proteins (either by modifying transcription or recycling) to prevent the harmful effect of the increased activity of Cv-c [250].

#### 4.2.2. Salivary Glands

The salivary glands (SG) of *Drosophila* are organs in the fly head secreting proteins that serve to fix the pupal case to a substrate. SG are formed by two elongated tubes of epithelial cells with a central lumen that are connected with a central duct that opens in the ventral side of the embryo. As PS, SG are relatively simple models of organogenesis, and their final shape is achieved without cell proliferation.

At about 4 hours of development, the primordia of the SG comprise about 100–120 cells in the ventral epidermis of parasegment 2 (the posterior part of the maxillary segment and the anterior part of the labial one) [258]. At about 6.5 h of development, two types of cells are distinguished in this primordium: secretory cells, located more dorsally and which secrete proteins to the lumen, and duct cells, which connect the secretory tubes with the mouth [259]. The secretory cells form two placodes in the ventral position of the labial segment. During stage 11 of embryogenesis the cells of the placodes undergo apical constriction, change their cell shape, and internalize sequentially, thus forming the tubes that elongate posteriorly into the thoracic region [259–261]. During this process there is no cell death or cell division [262–264].

The development of the SG depends on the activity of *Scr* because in *Scr* mutants the SG do not develop [259, 265]. In addition, the Hox cofactors Exd and Hth are also needed for this specification, as the glands are not formed in *exd* or *hth* mutants [266, 267]. The expression of *Scr* and *hth* disappears (and the Exd protein is located in the cytoplasm) before invagination of the secretory tubes, at stage 11 [267], and therefore subsequent development of SG is made without the input provided by *Scr*. Ectopic *Scr* expression throughout the embryo leads to new SG in two parasegments anterior to the labial one, parasegments 0 and 1 [259, 265, 268], but not posteriorly, since *Scr* activity is repressed in posterior segments by the genes *tea-shirt* and *Abd-B* [265]. These experiments demonstrate that *Scr* is necessary and sufficient to make SG but also that its activity is limited to the first stages of SG development.

The opposing activities of Dpp dorsally and the EGFR pathway ventrally confine secretory and duct cells to dorsal and ventral positions, respectively, within the segment. Secretory cells express genes like *forkhead* (*fkh*) and *huckebein* (*hkb*), whereas duct cells express genes like *trachealess* (*trh*) [259, 269]. In the complete absence of Dpp signaling, *trh* is expressed all over the segment [269] and all ectodermal parasegment 2 cells become SG; conversely, if Dpp activity is present throughout the embryo there are no SG [259, 269–272]. In a reciprocal way, the EGFR pathway blocks *fkh* expression ventrally and *fkh*, in turn, prevents the expression of duct cell genes [273, 274]. In the absence of EGFR signaling, duct cells become secretory cells, express genes like *fkh*, and repress genes like *trh* [273], normally confined to duct cells by *fkh* repression [269]. In this way, the opposing activities of the EGFR pathway and *fkh* distinguish duct cells from gland cells.

*(1) Primary Targets.* The early disappearance of *Scr* and its cofactors Exd and Hth indicates that the *Scr* target genes are likely to have a fundamental role in regulating salivary gland development. In fact, many of the *Scr* primary targets encode transcription factors whose expression in the SG, contrary to that of *Scr*, persists throughout development. Therefore, *Scr* dictates the formation of the SG but the cellular functions required to develop SG are implemented through the activity of *Scr* downstream genes, whose expression is maintained by cross-regulation and autoregulation. There are four primary targets of *Scr*: *fkh*, *hkb*, *Cyclic-AMP response element binding protein A* (*CrebA*), and *salivary gland-expressed bHLH* (*sage*) [259, 260, 265, 275–277].

*forkhead* (*fkh*). *fkh* encodes a FoxA winged-helix transcription factor orthologous to the mammalian hepatocyte nuclear factor 3*β* [278]. It is activated by *Scr* [259] but later on maintains its own expression [279] as well as the expression of other genes like *CrebA, senseless*, and *sage* [262, 280, 281]. In *fkh* mutants, salivary gland cells die by apoptosis [262].

*huckebein* (*hkb*). Another gene controlled by *Scr* is *hkb*, which codes for an *Sp1*/*Egr*-like transcription factor [282]. *hkb* expression is very dynamic and correlates with invagination timing of different cells [260], but its expression in SG is only transitory. In *hkb* mutants apical constriction and cell shape changes are normal, but the order in which the cells internalize is altered and the shape of the glands is no longer elongated [260].

*Cyclic-AMP Response Element Binding Protein A (CrebA).* This gene encodes a bZip transcription factor expressed at high levels in the SG throughout the larval period. *CrebA* is required for the secretion of proteins specific to the SG and for high levels of expression of genes of the early secretory pathway [280, 283]. *CrebA* is first activated by Scr-Hth-Exd and then maintained by *fkh* [262, 267, 280], and this regulation is direct [280]. Mutations in this gene reduce secretion, and its ectopic expression can induce the expression of secretory genes in other cell tubes like tracheae or salivary ducts [283].

*Salivary Gland-Expressed bHLH (sage). sage* codes for a bHLH protein that is only expressed in the SG [276, 277, 281, 284]. It is initially activated by *Scr* and *fkh* but maintained later on partly by autoregulation [281, 284]. *sage* regulates genes coding for proteins that modify other proteins in the secretory pathway or that travel through the secretory pathway (secreted proteins) [281, 284]. In *sage* mutants the SG cells die at stage 15-16, as revealed by the expression of the proapoptotic genes *rpr* and *hid* and by anti-cleaved caspase-3 staining [284].

*(2) Secondary Targets and Cellular Functions.* Many of the cellular functions required to make SG are regulated by *fkh*. In fact, an *in situ* hybridization analysis revealed that 59% of the genes expressed in SG show different levels of expression in *wildtype* and *fkh* mutant embryos [268]. The comparison of microarrays of *wildtype* and *fkh* mutant embryos showed that genes regulated by *fkh* encode proteins involved in many cellular functions [268]. *fkh* also maintains the expression of *Scr* primary targets like *CrebA* or *sage* [262, 275, 280, 281], thus explaining in part the pleiotropy of *fkh* mutations. However, contrary to what happens with *Scr*, ectopic *fkh* is not able to form extra SG [268]. Some of the cellular functions in which *fkh* is involved are invagination, tube maintenance, survival, and regulation of secreted cargo, membrane proteins, and enzymes.

*Invagination. fkh* is necessary for the apical constriction and cell shape changes of invaginating cells in the salivary placode [262, 278, 285]. This apical constriction, like that present in the formation of the ventral furrow [286, 287], requires the apical localization of myosin II and the activity of genes like *folded gastrulation*, *RhoGEF2*, and, similar to what happens in posterior spiracles [246], the RhoGTPase Rho1 [285]. Another protein that participates in RhoGTPase regulation in the SG is* 18-wheeler*, a Toll-like receptor protein [288]. *18-wheeler* expression in the SG depends on *Scr*, and in *18-wheeler* mutants invagination begins normally but the posterior sequential cell invagination observed in the wildtype is lost: too many cells invaginate at the same time and the whole process takes longer to be completed [289]. *18-wheeler* activates the RhoGTPase signaling pathway by inhibiting RhoGAPs like RhoGap5A and *RhoGap88C*/*cv-c*. Therefore, *Scr* regulates normal cell apical constriction and invagination of SG though *fkh* and *18-wheeler* controlling GTPase activity.

*Cell Death. fkh* is also required to prevent cell death. In *fkh* mutants there is ectopic expression of the proapoptotic genes *rpr* and *hid* and subsequent cell death [262, 290]. *fkh* regulates cell death partly through *senseless* (*sens*) and *sage*, whose absence also leads to cell death of secretory cells [277, 284].

*Secretion.* The SG form two secretory tubes that produce and secrete specific proteins [280]. This secretion requires the expression of *fkh*, which controls *CrebA* (first activated by *Scr*). *fkh* induces the expression of genes coding for proteins required for the translocation and targeting of specific secretory cargo (like mucins, larval cuticle proteins, and secreted enzymes) in the ER and of genes coding for proteins forming complexes in the endoplasmic reticulum and the Golgi, through *CrebA* regulation [275, 280]. Microarray studies show that *CrebA* may regulate some 400 genes, about half of which are related to secretion, targeting of proteins to the membrane, or involved in the secretory pathway and protein transport [283].

*Regulation of Lumen Size.* Two characterized targets of both *sage* and *fkh* are the prolyl-4-hydroxylase genes that encode two endoplasmic reticulum proteins, PH4*α*SG1 (SG1) and PH4*α*SG2 (SG2). These proteins hydroxylate proline residues in collagen and other secreted proteins [291]. SG1 and SG2 are needed to maintain the lumen size and when the function of these proteins is reduced, the altered secretory content in SG results in regions of tube dilatation and constriction [281].

Loss of *CrebA* reduces SG lumen size and content as well as the size and number of apically localized secretory vesicles [283]. *CrebA* has, therefore, a role in both secretion and tube maintenance. Rho1 also has a dual function; it is needed for salivary gland migration and for regulating the size of the lumen, and it does so through two different mechanisms [285, 292]: on one hand, Rho1 controls size of the lumen through regulation of the amount of phosphorylated Moesin at the apical membrane, which is important for the elongation of the apical domain [292]; on the other hand, it regulates cell rearrangements through controlling actin polymerization and distribution [292].

*Tube Elongation. hkb*, a primary target of *Scr*, is required for providing additional apical surface membrane (growth of apical membrane) for the elongation of invaginating cells [293]. In *hkb* mutants, the failure in providing additional apical surface results in reduced membrane length area and a failure of cell elongation. The end result is a change of shape of the organ, with dome-shape instead of elongated glands [260, 293]. *hkb* mediates tube elongation through two downstream targets that function to increase the apical membrane domain: *klarsicht* (*klar*) and *crb*. *klar* encodes a putative dynein-associated protein needed for directed movement of organelles [294, 295]; it mediates the polarized delivery of vesicles to the apical membrane through dynein, thus contributing to apical membrane growth and tube elongation. *crb* encodes a transmembrane protein that functions as an apical determinant [247, 248]; it is required in the apical region of the membranes to polarize cell shape changes during salivary gland invagination [293]. Thus, *crb* and *klar* control apical membrane elongation and delivery, respectively.

Tube elongation also requires the activity of *ribbon* (*rib*), which encodes a BTB/POZ domain-containing protein [296, 297]. *rib* mutant cells invaginate but do not complete migration posteriorly [297]. *rib* (in combination with the gene *lola like*) is necessary to promote *crb* expression and to downregulate activation (by phosphorylation) of Moesin, a protein that links *crb* to the cytoskeleton. The downregulation of the activity of Moesin and the upregulation of *crb* are necessary for the elongation and maintenance of the tube [298].

Another gene controlling tube elongation is *Rho1*.* Rho1* controls cell rearrangement and apical domain elongation by promoting actin polymerization at the basolateral membrane and limiting actin polymerization at the apical membrane. By controlling the distribution of F-actin in apical or basolateral membranes; *Rho1* regulates lumen size [292]. Rho1 regulates Moesin (in parallel to *rib*) and this control is necessary for the apical domain of the salivary gland cells to elongate and for the proximal gland lumen to narrow [292]. In *Rho1* mutants there are defects in lumen size and also in cell migration, although the mechanisms whereby *Rho1* governs these two processes are still unknown [285, 292, 299].

*Cell Migration.* The salivary cells invaginate, contact the visceral mesoderm, and then turn and migrate posteriorly using the circular visceral mesoderm, and perhaps other tissues, as a substrate [261, 263, 300]. This migration requires *Rho1* [285] and also the activity of the canonical Wg pathway through *Wnt-4*, the atypical Wnt receptor Derailed (Drl), and *wnt5*. In *drl* or *wnt5* mutants the direction of migration changes, curving towards the CNS. *drl* is expressed at the tip of the SG during migration, and its expression depends on *Scr* and *fkh* [301]. *Wnt4* is also needed in the second phase of migration, after contacting the visceral mesoderm, and in *Wnt-4* mutants the glands curve ventrally [301].

#### 4.2.3. Hox Genes and Organ Formation: Common Themes

The studies on PS and SG development unveil some characteristics, common to both processes, which may illuminate the way Hox genes create different organs.

1. The organ primordium is subdivided into groups of cells with independent control by Hox genes: in the PS, stigmatophore and spiracular chamber; in SG, secretory cells and duct cells. This subdivision of the task allows for independent development of the two structures. In spite of this independence, there must also be coordination of the two groups of cells in making the organ.
2. Hox genes control a limited number of primary targets that, in turn, regulate secondary genes that govern cellular tasks. It is not excluded, however, that in some cases there may be a double input on secondary targets, by primary targets and directly from Hox genes.
3. Cellular functions are in many cases regulated independently. For example, in the PS, mutations in cadherins disturb cell invagination but cell elongation is not affected [161]. However, some genes can regulate more than one cellular function. For instance, in the SG *CrebA* is needed for secretion and for regulation of lumen size [280, 283] and Rho1 for migration and for maintaining lumen size [285, 292, 299]. In the PS, *ems* regulates cell polarity through *crb*, the cytoskeleton through *Gef64C*, and adhesion through *cad88C* [161].
4. Few steps are needed in the genetic cascade to implement the basic cellular functions. This may have to do with the fact that organs like PS or SG have to develop quickly during embryogenesis, without cell proliferation. It may be that a higher number of steps are needed to make more complex organs during development.

## 5. Concluding Remarks

The strong transformations observed in some Hox mutations led to the definition of Hox genes as selector genes [40], implying that they are at the top of a gene hierarchy that determines size and pattern in a certain position of the body. Such morphological changes also spurred the interest in the identification of Hox targets, since their altered regulation would be responsible for distinguishing the wildtype structure from the mutated one. The detailed analysis of Hox mutations, however, reveals that in many cases Hox genes change previous genetic information that establishes general body plans. In this way, Hox genes simply modify, in a more evident or subtle way, signaling pathways and developmental routes at different times of development [91]. The fact that Hox genes assist in multiple modifications of gene activity, aiding the regulation of many genes in different tissues, led to the idea that Hox genes act in many cases as “micromanagers” that modify other genes' function to change morphologies rather than uniquely as master genes at the top of a genetic hierarchy [112]. Finally, the potential of Hox genes to construct organs with a minimal basic input of positional information would lead to consider them as “constructor” genes. The different views of Hox genes identify them, nevertheless, as major elements in determining structures in the anteroposterior axis of bilaterians.

The widespread role of Hox genes demands that they control many target genes and also raises the question of whether they regulate many of those directly or by intermediate targets. Detailed microarray studies and ChIP experiments suggested that Hox genes directly regulate many genes. The analysis of the formation of simple organs in *Drosophila*, however, argues for a direct regulation of a few genes, which in turn would control downstream realizators to carry out diverse cellular functions. The two views can be reconciled if Hox genes can in some instances simultaneously regulate one target both directly and through an intermediate. This double regulation would ensure a more efficient control of downstream genes and cellular functions. It is perhaps relevant also to consider that the analysis or organ formation by Hox genes awaits a detailed study of more complex structures, requiring processes like cell division. It may be that more elaborate regulations are needed in such cases.

The control of Hox downstream genes shows both a specific regulation of a certain target by a particular Hox protein and the existence of targets common to many Hox products. In general, there is a correspondence between the number of genes exclusively regulated by a Hox gene and the unique morphology or development it elicits. Because targets could change throughout development, their regulation by Hox proteins is very dynamic and the existence of particular or common targets will depend on the tissue and stage of development considered. Hox genes regulate targets that participate in different developmental routes, and this allows for an efficient coordination of tasks in order to produce a certain structure. This has implications not only for the development of a certain organism, but also for the evolution of different species. The mechanisms whereby Hox genes, in cooperation with other signals, implement morphological differences in evolution are being actively studied [196, 302–308], and these studies will cast light on how the different regulation of Hox targets has contributed to the morphological diversification of species.

## Figures and Tables

**Table 1:** Microarray expression profile and ChIP methods used to identify Hox targets. The Hox genes are studied in the wildtype, in Hox mutants, or after Hox ectopic expression. Modified from [47].

Organism	Hox gene	Tissue	Method	Reference
*Drosophila *	*lab *	Whole embryo	Microarrays	[53]
*Drosophila *	*Antp, Scr, *and* Ubx *	Imaginal discs	Microarrays	[54]
*Drosophila *	*Scr *	Leg imaginal discs	Microarrays	[61]
*Drosophila *	*Ubx *	Haltere and wing imaginal discs	Microarrays	[64]
*Drosophila *	*Ubx *	Haltere and wing imaginal discs	Microarrays	[66]
*Drosophila *	*Dfd, Antp, Scr, Ubx, abd-A, *and* Abd-B *	Whole embryo	Microarrays	[67]
*Drosophila *	*Ubx *	Haltere and wing imaginal discs	Microarrays	[73]
*Drosophila *	*Ubx *	Haltere imaginal disc	ChIP	[76]
*Drosophila *	*Ubx *	Haltere and third leg imaginal discs	ChIP	[77]
*Drosophila *	*Ubx *	Haltere imaginal disc	ChIP	[78]
*Drosophila *	*Dfd *	Whole embryo	ChIP	[79]
Mouse	*Hoxa13 *	Uterus and cervix	Microarrays	[52]
Mouse	*Hoxa11 *	Kidney cell lines	Microarrays	[55]
Mouse	*Hoxd10 *	Spinal cord	Microarrays	[56]
Mouse	*Hoxc8 *	Embryonic fibroblasts	Microarrays	[57]
Mouse	*Hoxa13 *	Embryonic fibroblasts	Microarrays	[58]
Mouse	*Hoxd cluster genes *	Limb and genital tissue	Microarrays	[59]
Mouse	*Hoxa1 *	Embryonic stem cells from blastocysts	Microarrays	[60]
Human	*Hoxa9, Hoxa10 *	Umbilical cord cells	Microarrays	[62]
Mouse	*Hoxa11, Hoxd11 *	Kidney	Microarrays	[63]
Mouse	*Hoxa1, Hoxb1 *	Whole embryos	Microarrays	[65]
Mouse	*Hoxa2, Hoxb1, Hoxb2* *Hoxb3, *and* Hoxd3 *	Rhombomeres 2–5	Microarrays	[69]
Mouse	*Hoxc13 *	Skin	Microarrays	[71]
Mouse	*Hoxa1 *	Rhombomeres 3–5	Microarrays	[72]
Mouse	*Hoxa13 *	NIH 3T3-derived embryonic fibroblasts	ChIP	[74]
Human	*Hoxd13 *	Humeral bone chondroblast cell line	ChIP	[75]
Mouse	*Hoxa2 *	Second branchial arch	ChIP	[80]
Zebrafish	*Hoxb1a *	Whole embryo	Microarrays	[68]
Zebrafish	*Hoxb1b *	Whole embryo	Microarrays	[70]
