# Erratum to “Hox Targets and Cellular Functions”

**DOI:** 10.1155/2014/529298

**Published:** 2014-04-13

**Authors:** Ernesto Sánchez-Herrero

**Affiliations:** Centro de Biología Molecular Severo Ochoa (CSIC-UAM), Nicolás Cabrera 1, Universidad Autónoma de Madrid, Cantoblanco, 28049 Madrid, Spain

Please add the following section.

